# Amended Vegetation Filters as Nature-Based Solutions for the Treatment of Pharmaceuticals: Infiltration Experiments Coupled to Reactive Transport Modelling

**DOI:** 10.3390/toxics12050334

**Published:** 2024-05-04

**Authors:** Raisa Gabriela Salvi-Taga, Raffaella Meffe, Virtudes Martínez-Hernández, Angel De Miguel Garcia, Irene De Bustamante

**Affiliations:** 1 Department of Geology, Geography and Environment, University of Alcala, 28802 Alcalá de Henares, Madrid, Spain; irene.bustamante@uah.es; 2 IMDEA Water Institute, 28805 Alcalá de Henares, Madrid, Spain; raffaella.meffe@imdea.org (R.M.); virtudes.martinez@imdea.org (V.M.-H.); 3 Wageningen Environmental Research (WEnR), Water and Food Team, Wageningen University and Research, 6708 Wageningen, The Netherlands; angel.demiguelgarcia@wur.nl

**Keywords:** vegetation filters, pharmaceutical removal, contaminants of emerging concern, soil, wastewater, column experiment, woodchips, soil amendments, flow and transport modelling

## Abstract

In small populations and scattered communities, wastewater treatment through vegetation filters (VFs), a nature-based solution, has proved to be feasible, especially for nutrient and organic matter removal. However, the presence of pharmaceuticals in wastewater and their potential to infiltrate through the vadose zone and reach groundwater is a drawback in the evaluation of VF performances. Soil amended with readily labile carbon sources, such as woodchips, enhances microbial activity and sorption processes, which could improve pharmaceutical attenuation in VFs. The present study aims to assess if woodchip amendments to a VF’s soil are able to abate concentrations of selected pharmaceuticals in the infiltrating water by quantitatively describing the occurring processes through reactive transport modelling. Thus, a column experiment using soil collected from an operating VF and poplar woodchips was conducted, alongside a column containing only soil used as reference. The pharmaceuticals acetaminophen, naproxen, atenolol, caffeine, carbamazepine, ketoprofen and sulfamethoxazole were applied daily to the column inlet, mimicking a real irrigation pattern and periodically measured in the effluent. Ketoprofen was the only injected pharmaceutical that reached the column outlet of both systems within the experimental timeframe. The absence of acetaminophen, atenolol, caffeine, carbamazepine, naproxen and sulfamethoxazole in both column outlets indicates that they were attenuated even without woodchips. However, the presence of 10,11-epoxy carbamazepine and atenolol acid as transformation products (TPs) suggests that incomplete degradation also occurs and that the effect of the amendment on the infiltration of TPs is compound-specific. Modelling allowed us to generate breakthrough curves of ketoprofen in both columns and to obtain transport parameters during infiltration. Woodchip-amended columns exhibited K_d_ and μ_w_ values from one to two orders of magnitude higher compared to soil column. This augmentation of sorption and biodegradation processes significantly enhanced the removal of ketoprofen to over 96%.

## 1. Introduction

Pharmaceuticals constitute a category of biologically active compounds engineered to interact with specific receptors within human or animal organisms. This diverse group encompasses a wide array of substances with varying chemical properties, likely leading to distinct behaviors within the environment. Due to demographic and epidemiological changes, the use of pharmaceuticals has increased significantly in OECD countries in the last decade [1] and, at present, over 2000 active pharmaceutical ingredients are prescribed on a global scale [2]. Pharmaceuticals, along with their transformation products (TPs), have been detected in raw and treated wastewater; surface and groundwater; soil; sediments; and even crops [3,4,5,6,7,8,9,10,11,12,13,14]. Even at the typically observed concentrations (in the range of ng L^−1^ or μg L^−1^), these substances may elicit known or suspected adverse effects on aquatic and terrestrial ecosystems [15,16,17,18,19]. These effects can, in turn, have repercussions for human health [15,16,17,18,19], which may cause resistance to antimicrobial activity, leading to enhanced virulence, increased mortality [20,21,22] and endocrine disruption disorders, such as alteration of hormone functions, retarded maturity, decreased fertility and thyroid function abnormalities [23,24]. The extensive presence and potential ecological and human health implications of pharmaceuticals in the environment underscore the importance of thorough investigation and management of these compounds.

Effluents originating from wastewater treatment plants (WWTPs) are recognized as significant contributors to the presence of pharmaceuticals and their TPs in the environment, primarily due to incomplete removal during treatment processes [25,26]. The concern related to the presence of pharmaceuticals in WWTP effluents is demonstrated by the update to the Urban Wastewater Treatment Directive (UWWTD) (Council Directive 91/271/EEC) that is currently underway for approval by the European Parliament. Such an initiative emphasizes the necessity of removing specific contaminants of emerging concern, notably pharmaceuticals, from urban wastewater [27]. To address the persistent challenge of pharmaceuticals and TPs entering the environment, various wastewater treatment techniques have been explored to enhance their removal [28,29,30,31]. Tertiary wastewater treatment technologies, such as activated carbon, advanced oxidation processes (AOPs) and reverse osmosis, have demonstrated remarkable effectiveness in eliminating targeted pharmaceuticals, achieving removal rates exceeding 90% [29]. However, these more advanced systems are not affordable, especially for small municipalities, as they require higher installation and maintenance costs and personnel with adequate training for their operation.

On the other hand, non-conventional nature-based wastewater treatment systems, such as vegetation filters (VFs), are considered a viable alternative for small populations and scattered communities, due to their low cost for implementation, limited energy use and reduced operation and maintenance requirements [32,33]. A VF consists of a land area, usually covered by a forestry plantation, to which pre-treated wastewater, mainly urban-type, is applied with the aim of removing wastewater-originating contaminants through natural attenuation mechanisms by the combined action of soil, plants and microorganisms [33,34]. The main processes contributing to the attenuation of contaminants in VFs are sorption onto the soil, biodegradation, chemical precipitation and plant uptake [32,35]. Although a simple operation and maintenance is required, a system failure could result in groundwater pollution, and a good design procedure is essential for the success of this technology [33]. Martínez-Hernández et al. [32] report on removals of more than 90% through VFs of target pharmaceuticals and TPs, including antibiotics, analgesics, anticonvulsants, antidepressants and β-adrenergic blockers, whereas lower attenuation (~55%) was obtained for the non-steroidal anti-inflammatory (NSAID) ketoprofen, revealing its more recalcitrant nature.

In this context, soil amended using readily labile carbon sources to enhance microbial activity and sorption processes can be considered a feasible solution for ameliorating VF performance [36,37]. Using wood as a carbon source offers numerous benefits, such as its widespread availability at an affordable price, its elevated C:N ratio and its exceptional durability [38]. Moreover, when integrated into a VF, carbonaceous material can be directly produced by the plants in the treatment system. More labile carbon sources, such as corn stalks and straw, can deliver higher removal rates. However, they are typically consumed more quickly, necessitating frequent material replenishment [39]. Furthermore, other carbon sources, such as biochar, can be subject to additional treatment options, including the combination with catalysts such as zerovalent iron to enhance the oxidation of organic compounds using persulfate in soil [40]. However, such treatments require additional energy input and associated costs that should considered.

Column experiments coupled with numerical modelling are useful tools to rapidly test soil amendments prior to its incorporation in real VFs and to delineate the dynamics of water flow and contaminant transport within porous media providing predictive insights into the fate and migration of contaminants. The predominant focus in pharmaceuticals of recent research has leaned towards experiments conducted under saturated conditions particularly to forecast their transport in aquifers and natural soils, as well as during processes such as aquifer recharge, agriculture and urban irrigation and soil aquifer treatment [41,42,43,44,45,46,47]. In contrast, investigations carried out under unsaturated conditions are comparatively limited, and even scarcer are studies conducted under unsaturated conditions that employ soil amendments as a treatment approach for pharmaceutical removal. Specifically, only a few studies have explored the use of sustainable materials like biochar, woodchips, biosolids, clay and zeolite as soil amendments for this purpose [37,48,49,50,51,52]. Nevertheless, none of these studies have integrated their experimental work with comprehensive transport and flow modelling.

To overcome this research gap, Meffe et al. [36] have carried out column tests aimed to investigate if soil amendments with woodchips using different configuration enhance the treatment of wastewater-originated nutrients and organic matter. Successively, more experiments were carried out to expand this research using the same experimental set-up, to analyze the attenuation of the pharmaceuticals acetaminophen and naproxen (analgesics), atenolol (β-adrenergic blocker), caffeine (stimulant), carbamazepine (anticonvulsant), ketoprofen (NSAID) and sulfamethoxazole (antibiotic) and TP formation (atenolol acid and carbamazepine epoxide). The pharmaceuticals were selected based on the results of a 2-year monitoring of a pilot poplar VF receiving urban wastewater at the R&D&I Centre of Carrión de los Céspedes (Seville, Spain) [32].

The objectives of the present work consist of (i) assessing whether woodchip amendments to the soil of a VF are able to abate pharmaceutical concentrations in the infiltrating water, (ii) quantifying and comparing the reactive processes to which the pharmaceuticals are subjected in the unsaturated zone and in the woodchips and (iii) analyzing qualitatively the formation of pharmaceutical TPs in the presence of woodchips.

## 2. Materials and Methods

### 2.1. Column Experiments

**Figure 1:**
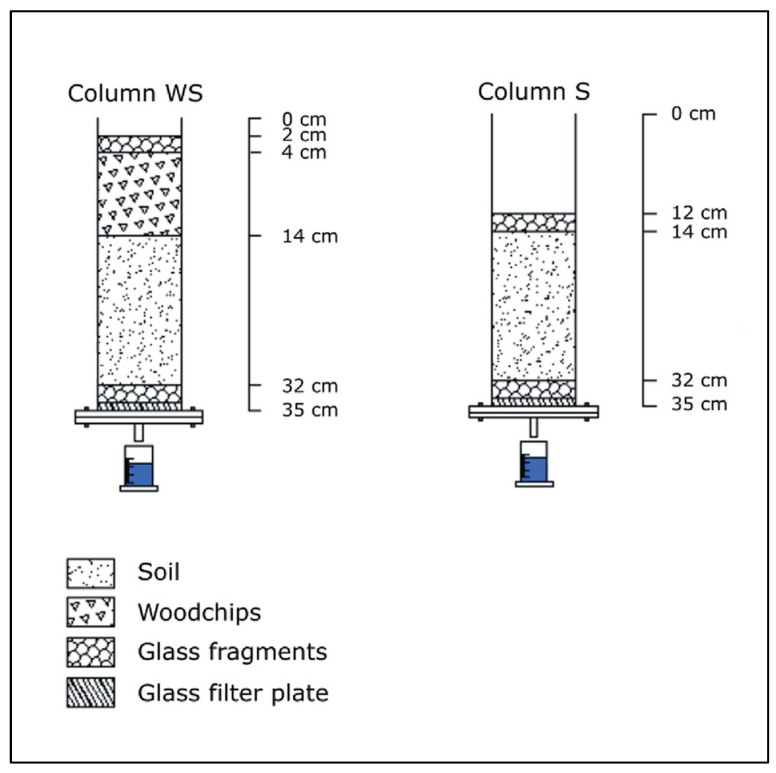
Schematic representation of the column experimental set-up (edited from Meffe et al. [36]).

The experiments investigating pharmaceutical transport were performed using two of the original columns used by Meffe et al. [36]: (i) Column S, which contained only soil (18 cm thickness) and was therefore used as a reference, and (ii) Column WS, with a 10 cm layer of poplar woodchips placed over the soil surface (total thickness of 28 cm) (Figure 1). The assays were run simultaneously and lasted 137 days for Column S and 143 days for Column WS.

The columns were saturated by an upward flow with synthetic wastewater (SWW), which mimicked real wastewater applied to the VF in Carrión de los Céspedes (Seville, Spain) characterized by a high nutrient load (average N_T_ 838 kg ha^−1^ and P_T_ 87 kg ha^−1^) [34,36]. The use of SWW ensures a constant chemical composition at the columns’ inlet. The SWW was produced by dissolving the following reagents (purity > 95.0%) in tap water: NaCl (0.100 g L^−1^), MgSO_4_ (0.055 g L^−1^), K_2_HPO_4_ (0.050 g L^−1^), (NH_4_)_2_CO_3_ (0.650 g L^−1^), KCl (0.050 g L^−1^), peptone (0.075 g L^−1^), meat extract (0.175 g L^−1^) and 1 mg L^−1^ of the studied pharmaceuticals. Once prepared, the SWW was purged with nitrogen gas (N_2_) until the dissolved oxygen concentration was below 1.5 mg L^−1^. The compositions of the SWW and the original wastewater are detailed in Meffe et al. [36].

Once saturated, the columns were weighed to obtain the water content and afterwards they were allowed to drain by gravity until the effluent flow completely stopped. Daily additions of 50 mL of SWW (corresponding to 6.4 L m^−2^) were performed manually, with the exception of weekends when influent solution was provided through an automated peristaltic pump. Once the equilibrium in the effluents was reached (constant pH, electrical conductivity- EC and redox potential), a tracer test was performed in both systems to obtain the columns’ hydraulic parameters and estimate the residence time. To this end, a 10 g L^−1^ sodium chloride (NaCl) solution was injected as a single pulse and EC and Cl^−^ concentrations were monitored in the effluents for 46 days [36]. Approximately 3 months from the beginning of the experiment, 50 mL (6.4 L m^−2^) of a SWW containing a concentration of 1 mg L^−1^ of ketoprofen, acetaminophen, atenolol, caffeine, carbamazepine, naproxen and sulfamethoxazole was applied daily. The inlet concentration of 1 mg L^−1^ was selected to increase the probability of achieving quantifiable concentrations of pharmaceuticals and TPs at the column outlets. During the weekend, SWW was maintained under dark conditions, continuously agitated to keep organic concentration input constant and provided to the columns through a peristaltic pump.

### 2.2. Analytical Method

Daily column effluent samples were collected, their weight recorded and pH, EC and redox potential measured. Pharmaceutical concentrations in the effluent of both columns were measured twice a week during the 62 days following the first injection. The formation of TPs of atenolol (atenolol acid) and carbamazepine (carbamazepine epoxide) was also monitored throughout the experiment. Prior to the analysis, samples were spiked with sodium azide to avoid pharmaceutical biodegradation and filtered through 0.45 µm PTFE membranes. If the analysis were not immediately carried out, the samples were preserved and stored, for no longer than three weeks, at −21 °C.

The concentration of pharmaceuticals in the liquid phase was analyzed in the laboratories of the IMDEA Water Institute (Madrid, Spain). The analysis involved the direct injection of 50 μL samples into LC-MS TripleTOF 5600 system (AB SCIEX, Concord, ON, Canada) connected to an HPLC system with an electrospray interface (ESI). The method quantification limits (MQLs) were determined experimentally by the injection of spiked blank water samples. For assessing matrix effects, a comparative analysis of the regression lines in matrix-matched samples and standards was executed. MQLs were 0.5 μg L^−1^ for acetaminophen, 0.1 μg L^−1^ for atenolol, 0.5 μg L^−1^ for caffeine, 0.1 μg L^−1^ for carbamazepine, 0.2 μg L^−1^ for ketoprofen, 1.0 μg L^−1^ for naproxen, 0.1 μg L^−1^ for sulfamethoxazole, 0.01 μg L^−1^ for atenolol acid and 0.5 μg L^−1^ for carbamazepine epoxide. Prior to analysis, all samples were spiked with a mixture of surrogate standards (terbutylazine-D5 and ^13^C-caffeine) in acetonitrile (Sigma-Aldrich). All chemicals (pharmaceutical standards and reagents) used were of analytical grade and purchased from Sigma-Aldrich (Madrid, Spain) (purity > 97%).

### 2.3. Numerical Modelling

Steady-state one-dimensional flow and solute transport models were set up for both columns using Hydrus-1D version 4.17, a software program that simulates the unidimensional water flow dynamics, as well as the solute and heat transport in the vadose zone [53,54]. The software solves the Richards equation for variably saturated water flow through a porous system and the solute transport is described through the classical Fickian-based convection–dispersion equation [53,55].

#### 2.3.1. Governing Water Flow and Tracer Transport Formulations

The equation that describes the unidimensional uniform (equilibrium) water flow through an unsaturated porous medium is the modified form of the Richards equation, as defined below [53]:

(1) $$\frac{\partial \theta }{\partial t}=\frac{\partial }{\partial z}\left[K\left(\frac{\partial h}{\partial z}+\cos\alpha_{d}\right)\right]-S$$

where h is the pressure head [L], θ is the volumetric water content [L^3^ L^−3^]; t is time [T]; z is the spatial coordinate [L]; S is the sink term [L^3^ L^−3^ T^−1^]; $\alpha_{d}$ is the angle between the flow direction and the vertical axis ($\alpha_{d}$ = 0 for vertical flows) and K is the unsaturated hydraulic conductivity function [L T^−1^].

When necessary, the equilibrium flow model can be modified to take into account the microporosity of the soil and/or material where the water is considered to be immobile (nonequilibrium physical model) [55]. Indeed, in dual-porosity systems, the material intra-aggregate pores consisting of immobile water pockets can exchange, retain and store water, but convective flow is not permitted [53]. This assumption divides the liquid phase into mobile (inter-aggregate), $\theta_{mo}$ and immobile (intra-aggregate), $\theta_{im}$ [L^3^ L^−3^] [53]:

(2) $$\theta =\theta_{mo}+\theta_{im}$$

The formulations that describe the dual-porosity type flow is based on a mixed formulation of the Richards equation for the water flow in the macropores, combined with a mass balance equation for the dynamics in the intra-aggregate pores [53,55], as follows:

(3) $$\frac{\partial \theta_{mo}}{\partial t}=\frac{\partial }{\partial z}\left[K\left(\frac{\partial h}{\partial z}+\cos\alpha_{d}\right)\right]-S_{mo}-\Gamma_{w}$$

(4) $$\frac{\partial \theta_{im}}{\partial t}=-S_{im}-\Gamma_{w}$$

(5) $$\Gamma_{w}=\frac{\partial \theta_{im}}{\partial t}=\omega \left({S_{e}}^{mo}-{S_{e}}^{im}\right)$$

where $S_{mo}$ and $S_{im}$ are sink terms for both regions [T^−1^], $\Gamma_{w}$ is the transfer rate for water from the inter- to intra-aggregate pores [T^−1^], ω is the first-order rate mass transfer coefficient [T^−1^] and ${S_{e}}^{mo}$ and ${S_{e}}^{im}$ are effective fluid saturation of the mobile and immobile regions, respectively.

With respect to the tracer, the following equation, based on Fick’s Law, describes the non-reactive transport through an unsaturated porous media [54]:

(6) $$\frac{\partial \theta c}{\partial t}=\frac{\partial }{\partial z}\left(\theta D^{w}\frac{\partial c}{\partial z}\right)-\frac{\partial (J_{w}c)}{\partial z}$$

where c is the solute concentration in the liquid phase [M L^−3^], $J_{w}$ is the water flux [L T^−1^], and $D^{w}$ is the hydrodynamic dispersion [L^2^ T^−1^] defined as [53,56]:

(7) $${\theta D}^{w}=\alpha_{L}\left|J_{w}\right|+\theta D_{w}\tau_{w}$$

where $\alpha_{L}$ is the longitudinal dispersivity [L], $D_{w}$ is the molecular diffusion coefficient in free water [L^2^ T^−1^], and $\tau_{w}$ is the tortuosity factor in the liquid phase [-]. $\tau_{w}$ is calculated as a function of the water content, according to the Millington and Quirk relationship [57]:

(8) $$\tau_{w}=\frac{\theta^{7/3}}{{\theta_{s}}^{2}}$$

where $\theta_{s}$ is the saturated volumetric water content [L^3^ L^−3^].

#### 2.3.2. Reactive Solute Transport Formulations

The pharmaceutical reactive transport model developed in this study takes into account sorption and biodegradation as the principal attenuation processes. Given that pharmaceutical inlet concentration in both columns was lower than 10^−5^ M, linear and instantaneous sorption between the solid and aqueous phases was considered from the beginning [58]. On the other hand, the first-order equation was used to account for biodegradation ruling out the occurrence of any lag phase in microbially mediated processes. Indeed, the soil used in the experiments was exposed for more than three years to pre-treated wastewater containing target pharmaceuticals. The equations describing such processes are the following [53,54]:

(9) $$\frac{\partial \theta c}{\partial t}+\frac{\partial \rho s}{\partial t}=\frac{\partial }{\partial z}\left(\theta D^{w}\frac{\partial c}{\partial z}\right)-\frac{\partial J_{W}c}{\partial z}-\mu_{w}\frac{\partial \theta c}{\partial z}$$

(10) $$s=K_{d}c$$

where ρ is the bulk density [M L^−3^], s is the solute concentration in the solid phase [M M^−1^], $\mu_{w}$ is the first-order kinetic removal rate constant [T^−1^], and $K_{d}$ is the sorption distribution coefficient [L^3^ M^−1^].

#### 2.3.3. Model Set-Up

Model parameters are presented in Table 1. Time and space discretion was performed automatically, maintaining Courant and Peclet numbers below 1 and 2, respectively [54]. Furthermore, to refine the model mesh, the maximum number of nodes existing in Hydrus 1D was considered for Column WS and S. A finer discretization was applied at the upper and bottom part of both columns and at the interface between the soil and woodchip layers in Column WS.

The infiltration of water through Column S and Columns WS was initially simulated selecting the equilibrium water flow. In this case, the van Genuchten–Mualem model with no hysteresis was used to describe soil hydraulic properties [60]. Successively, the standard equilibrium water flow model was upgraded with a dual-porosity model, applied exclusively in the model domain occupied by the woodchip layer, in order to obtain a more accurate representation of the water flow dynamics within this specific layer.

Based on texture data of the soil used in the experiments (Table 1), the soil hydraulic parameters were estimated by hierarchical pedotransfer functions using the Rosetta program included in Hydrus-1D [61].

For both columns, the initial pressure heads used as input parameters for transient simulations were obtained by first performing runs under steady state conditions. In this sense, the drainage of the initially saturated columns (h = 0) was simulated during an interval of time long enough (6 days) to achieve constant values of pressure heads along the soil and soil + woodchip profile.

The upper boundary condition (BC) for water flow simulations was defined as the atmospheric BC with the surface layer (max. water height of 0.65 cm). The daily addition of SWW was simulated by applying a flux of 0.6366 cm s^−1^ and repeating this pattern every 24 h. Between applied SWW and water recollected at the outlet, there was a difference in volume (average of 21.5% and SD of 13.1%) that was interpreted as a consequence of evaporation occurring at the soil or woodchip surface. For this reason, a daily average evaporation rate was applied to take into account the loss of water.

On the other hand, the seepage face BC, which was specific to column experiments, was set at the lower boundary of the solution domain.

For transport simulation, applied concentrations of both tracer and target pharmaceuticals (see Table 1) were simulated at the column inlet (Concentration Flux BC). At the column outlet, a zero-concentration gradient was defined for both models as the lower BC. Furthermore, for both columns, the solute initial conditions were specified in terms of liquid concentration (mass of solute/volume of water).

#### 2.3.4. Model Calibration and Adjustments

The flow models have been calibrated using the water volumes collected daily at the columns’ outlets. The parameters K_s_ and α_L_ of the soil layer were first calibrated through the non-reactive transport model developed for Column S. Successively, obtained values were implemented in the Column WS solution domain occupied by soil to allow for calibration of the same parameters in the woodchip domain. The dual-porosity model parameter ω (see Table 1) and the corresponding woodchip hydraulic properties parameters (θ_r_, α and n) were included in this calibration. Finally, the woodchip θ_s_ measured gravimetrically (0.85 cm^3^ cm^−3^) was partitioned between the mobile and immobile water content (θ_smo_ and θ_sim_).

A similar modelling approach was used for the reactive transport modelling. Column S, as a reference column, was used to obtain the reactive transport parameters *K_d_* and μ_w_ for the soil layer. A variation of ±20% was applied to the obtained *K_d_* and μ_w_ to analyze the impact of these parameters on the shape and characteristics of the breakthrough curve.

Subsequently, *K_d_*_,_ calibrated in Column S, was applied in the Column WS solution domain occupied by the soil layer, allowing for calibration of *K_d_* in the woodchip layer and μ_w_ in both layers/materials. Considering that interactions between soil and woodchip layer in terms of biodegradation processes are difficult to predict, different scenarios were simulated to calibrate μ_w_ in both materials in Column WS. Scenario 1 considers similar μ_w_ in both soil and woodchip layers; Scenario 2 simulates different μ_w_ between the layers; Scenario 3 and Scenario 4 do not simulate any degradation in the woodchip and soil layers, respectively, and Scenario 5 considers the same μ_w_ calibrated in Column S, also applying it to the soil layer of Column WS.

The selection of the best fit is based on the maximization of the regression coefficient between observed and simulated data and on the minimization of the 95% confidence interval. Values of estimated parameters were introduced in the model as initial guess of inverse calibration until achieving the optimal fit of observed data.

The measure of goodness-of-fit was calculated though the root mean squared error (RMSE) and the coefficient of determination ($R^{2}$), as follows:

(11) $$RMSE=\sqrt{\frac{1}{n}\sum_{i=1}^{n}{\left(\hat{y_{i}}-y_{i}\right)}^{2}}$$

(12) $$R^{2}=\frac{{\left[\sum \hat{y_{i}}y_{i}-\sum \hat{y_{i}}\sum y_{i}\right]}^{2}}{\left[\sum {\hat{y_{i}}}^{2}-{\left(\sum \hat{y_{i}}\right)}^{2}\right]\left[\sum {y_{i}}^{2}-{\left(\sum y_{i}\right)}^{2}\right]}$$

where $y_{i}$ is the simulated data, $\hat{y_{i}}$ is the experimental data (observed), and n is the data set number.

### 2.4. Sensitivity Analysis

With the objective of analyzing the effect of input parameter uncertainty in the results obtained by modelling the experimental data of Column WS, a sensitivity study was performed. The parameter perturbation method using a single normalized sensitivity coefficient (Equations (13) and (14)) [62] was applied to the following hydraulic parameters from the woodchip layer: θ_sIm_, θ_smo_, α, θ_rmo_, θ_rIm_, n, l and ω.

The values of these parameters varied within a range of ±15% to ±25%, and the effects of these variations were analyzed in terms of changes of simulated concentration in the column effluent.

(13) $$\chi_{k}=\frac{\frac{\left(S\left(P_{k}+∆P_{k}\right)-S\left(P_{k}\right)\right)}{S\left(P_{k}\right)}}{\frac{∆P_{k}}{P_{k}}}$$

(14) $$S=\sum_{i=1}^{n}\frac{{\left(C_{{sim}_{i}}-C_{{mea}_{i}}\right)}^{2}}{N}$$

where $\chi_{k}$ is the normalized sensitivity coefficient, $S\left(P_{k}\right)$ is the sum-of-square objective function of the base case, $S\left(P_{k}+∆P_{k}\right)$ is the sum-of-square objective function of the altered case ($P_{k}$ is varied to $∆P_{k}$) and $C_{{sim}_{i}}$ and $C_{{mea}_{i}}$ are, respectively, the simulated and experimental pharmaceutical concentrations in the Column WS effluent.

## 3. Results

### 3.1. Water Flow, Woodchip Hydraulic Parameters and Conservative Transport

As aforementioned, the flow models have been calibrated using the daily water volumes flowing out from the columns. Table 2 presents experimental and simulated cumulative water volumes. The single-porosity (physical equilibrium) model developed for Column S is able to reproduce water volumes with a minimal difference with observed data (RMSE of 4.59 mL). Conversely, the single-porosity approach performs with less accuracy in reproducing the water flow through Column WS. In this case, the RMSE in terms of cumulative water volumes is almost halved when a dual-porosity model is applied.

**Figure 2:**
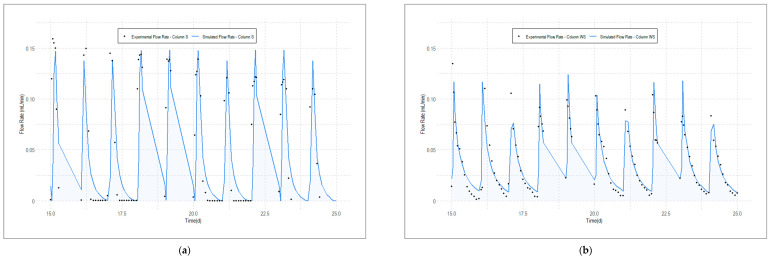
(**a**) Experimental and corresponding simulated average outflow flow rates from Column S (single-porosity model) (RMSE: 0.043 mL/min); (**b**) experimental and corresponding simulated average outflow flow rates from Column WS (dual-porosity model) (RMSE: 0.015 mL/min).

Figure 2 presents experimental and simulated average flow rates when a single-porosity (Column S) and a dual-porosity (Columns WS) approaches are applied. The fluctuation in the average outflow flow rate for both columns displays a nearly consistent pattern, characterized by daily cyclic periods of sharp increase just after the SWW, followed by a swift decline during the drying phase between the irrigation intervals. The main difference between Column S and Column WS is the tailing shape in the flow rate of the latter as a consequence of the continue supply from the woodchip layer once the main wetting front left the column.

The simulated and experimental average flow rate data from both columns reveals a satisfactory level of concurrence, with a RMSE of 0.043 mL/min for Column S and 0.015 mL/min for Column WS. Similarly to what was observed for the cumulative water volume flowing from the column, the RMSE is halved with the dual-porosity model (0.030 mL/min vs. 0.015 mL/min). The figure presenting the Column WS average flow rates simulated through the single-porosity model can be found in the Supplementary Materials Section, Figure S1.

**Figure 3:**
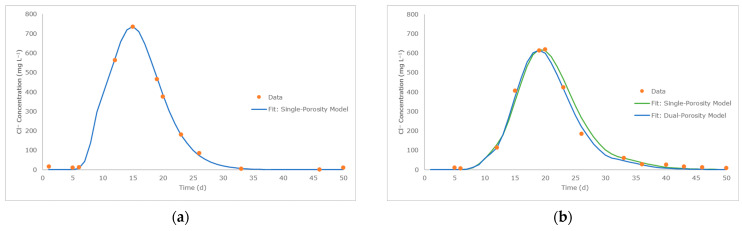
Simulated breakthrough curves of Cl^−^: (**a**) Column S (single-porosity model, R^2^ = 0.999); (**b**) Column WS (single-porosity model, R^2^ = 0.981); dual-porosity model, R^2^ = 0.995).

The simulation of the tracer breakthrough curves for both columns are presented in Figure 3. Excellent fits of experimental data are obtained in all cases (R^2^ of 0.999 and 0.981–0.995 for Columns S and WS, respectively). The breakthrough curves derived from both the single- and dual-porosity models for Column WS exhibit a noticeable similarity. However, the simulated water flow dynamics of the two models differ significantly. Notably, the dual-porosity model provides a more accurate reproduction of the experimental results, as corroborated by the higher R^2^ (Figure 2) and lower RMSEs of the average outflow flow rate and simulated cumulative water volume (Supplementary Materials Section and Table 2, respectively). Such results confirm what was reported by Subroy et al. [63] regarding the dual domain as an approach able to simulate the pore system of woodchips.

The Cl^−^ concentration peak of the breakthrough curves gives an estimation of the average residence times, which is 15 days for Column S and 19 days for Column WS. The higher residence time for Column WS is the result of the increased water content due to the presence of woodchip layer (saturated water content of 600.27 mL for Column S and 970.95 mL for Column WS), as well as the additional 10 cm of length of Column WS.

The inverse fitting of measured Cl^−^ concentrations provides the K_s_ and α_L_ of the Column S and WS soil and woodchip layers (Table 3), the woodchip hydraulic parameters ω, θ_rmo_, θ_rIm_, α, n, θ_smo_ and θ_sIm_ (θ_smo_ + θ_sIm_ = 0.85 cm^3^ cm^−3^) (Table 4), as well as the solute (Cl^−^) molecular diffusion coefficient in free water (D_w_).

K_s_ obtained for the soil layer is consistent with the tabulated values for sandy loam soils [64]. Similarly, the K_s_ value of 0.0218 cm s^−1^ for the woodchip layer is within the range of 0.0142–0.18 cm s^−1^ reported by Driel et al. [65] and Subroy et al. [63] for this material. The α_L_ is highly dependent on the experiment scale [66] and the fitted value for the soil layer is among the interval commonly obtained for soil column tests [67]. The α_L_ of the woodchip layer is one order of magnitude larger than that of the soil layer, which is consistent with findings from Lynn et al. [68].

Numerical modelling of water flow and contaminant transport through woodchip layers are rather scarce in the literature. Those available have been developed to predict the behavior of woodchip-based treatment systems such as denitrification bioreactors [69,70,71,72,73] and to forecast the generation of leachate from woodchip stockpiles [63,74]. None of the available modelling studies have investigated water infiltration and solute transport through woodchips with an underlying soil that also conditions the hydraulics of the entire system. The values obtained for θ_smo_ (0.273 cm^3^ cm^−3^) and θ_sIm_ (0.577 cm^3^ cm^−3^) indicate that a significant portion of the woodchip pores are intra-aggregate immobile pores, consistent with the results of Subroy et al. [63]. Specifically, calibrated data suggest that approximately 67.9% of the woodchip pores possess the ability to exchange, retain and store water. This finding also coincides with the observed tailing in flow rates (Figure 2) and tensiometer data showed in Meffe et al. [36], confirming and quantifying the capacity of the woodchip layer to retain water and release it once the main wetting front has passed through Column WS. Furthermore, the optimized values obtained for θ_smo_ and θ_sIm_ are similar to those reported by Jaynes et al. [69]. The low value of ω (3.54 × 10^−7^ s^−1^) suggests that the water exchange between the mobile and immobile pore domains is a slow process.

In the context of contaminant transport modelling, molecular diffusion, is usually disregarded, as it is inherently a very slow process and relegated to a secondary consideration when compared to the more dominant mechanisms of advection and dispersion [75]. However, under the experimental conditions in which flow rates transiently approach zero (see Figure 2) and the water flow is sluggish, the role of advection becomes momentarily limited and molecular diffusion predominates in the contaminant transport. The molecular diffusion in free water (D_w_) is in the order of 10^−5^ cm^2^ s^−1^ for most chemicals in the liquid phase and is temperature dependent [76]. Fitting D_w_ for Cl^−^ in the tracer tests of Column S and WS, respectively, resulted in values of 1.71 × 10^−5^ and 2.98 × 10^−5^ cm^2^ s^−1^, compatible with the 2.03 × 10^−5^ cm^2^ s^−1^ at 25 °C reported by Li et al. [77].

### 3.2. Reactive Transport through the Soil Layer

**Figure 4:**
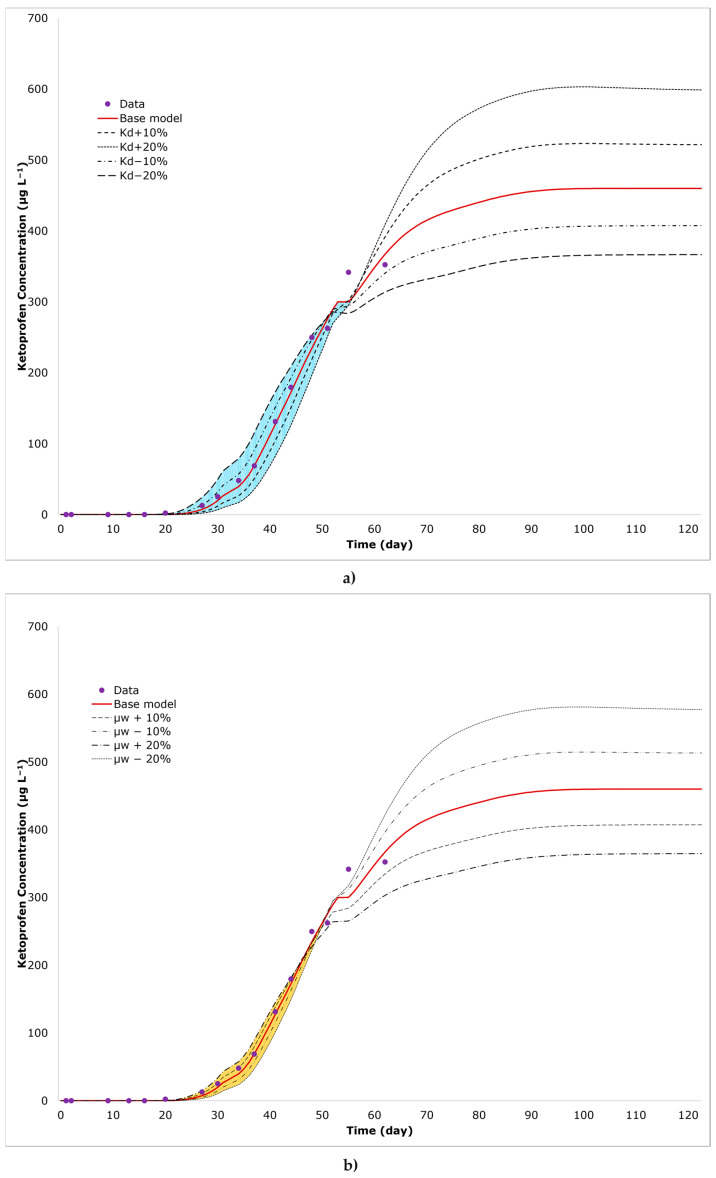
Measured ketoprofen breakthrough curve and corresponding fit for Columns S. The solid red line represents the base model fit (R^2^ = 0.991), and the dashed lines represent the simulation results, applying a variation of ±10–20% in *K_d_* (**a**) and µ_w_ (**b**). The blue and yellow areas correspond to the intersect between the ±20% *K_d_* and µ_w_ curves, respectively.

The anti-inflammatory ketoprofen was the only injected pharmaceutical that reached the column outlet of both systems within the experimental time (62 days). Modelling results clarify that observed concentrations did not reach a plateau, indicating incomplete breakthrough curves (Figure 4). Despite the incomplete set of data, the modelling of the breakthrough curves with the support of scenario simulations allows us to tackle the shortcoming of qualitative interpretations, providing reliable ranges for reactive transport parameters (as shown in Table 5 and Table 6).

Regarding Column S, transport of ketoprofen can be well described with an equilibrium sorption model and a first-order degradation model, obtaining an excellent fit of experimental data (R^2^ = 0.991). By varying the calibrated K_d_ by ±20%, a reasonable fit of observed data is still maintained for *K_d_* variations of up ±10% (R^2^ = 0.9790–0.9859) (Table 5). However, it becomes apparent that this level of fit does not persist when K_d_ variations exceed ±10%. For *K_d_* variations greater than ±10%, R^2^ drops below 0.970, indicating a less satisfactory alignment between the model and observed data. Consequently, the reliable range for K_d_ values in the soil layer lies between 0.434 to 0.530 L kg^⁻1^.

The variation of *K_d_* has a greater impact on the initial stage of the breakthrough curve (arrival time). On the other hand, µ_w_ has a more pronounced effect on the region occupied by the curve plateau, where experimental data are not available. Therefore, a wider range of µ_w_ (±20%) still yields R^2^ values ≥ 0.970 (Table 5). According to model simulations, the acceptable range of degradation values for the soil layer falls between 0.0584 and 0.0875 d⁻^1^.

Fitted *K_d_* in the soil layer allows for the calculation of a retardation factor in the range of 2.55–2.89. The inverse fitting also describes the occurrence of a very limited ketoprofen degradation in Column S, mirroring the persistence of this anti-inflammatory (Table 5) during infiltration through the soil. Indeed, the low values of *K_d_* and μ_w_ are consistent with the results from studies from Breuer et al., Kiekak et al., Styszko et al. and Xu et al. [42,78,79,80], indicating that the processes of sorption and degradation of this compound are low or limited in soil or sediment and various orders of magnitude lower when compared to other pharmaceuticals, such as diclofenac, antipyrine, atenolol, carbamazepine and sulfamethoxazole. The persistence of ketoprofen observed during its infiltration in the unsaturated zone at a laboratory scale aligns with the limited removal (55.4%) of this compound reported in our previous VF pilot-scale study [32] in Carrión de los Céspedes (Seville, Spain).

### 3.3. Effects of Using Woodchips as Soil Amendments in Vegetation Filters on Flow and Contaminant Attenuation

**Figure 5:**
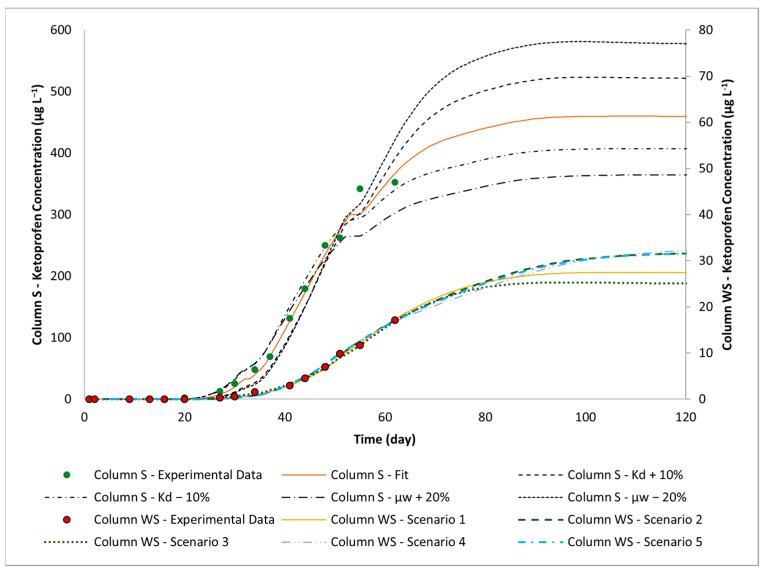
Comparison between measured ketoprofen breakthrough curve and corresponding fits for Column S and WS. The secondary y axis corresponds to observed and fitted data of Column WS.

Satisfactory results are obtained for the five ketoprofen reactive transport modelling scenarios carried out for Column WS (R^2^ between 0.995 and 0.997, refer to Figure 5) using the equilibrium sorption and a first-order degradation rate. In all cases, simulated K_d_ in the woodchip layer (from 11.20 to 15.41 L kg^−1^) is one order of magnitude higher than that obtained for soil (0.43 to 0.53 L Kg^−1^), indicating the ability of the carbonaceous material to retain organic molecules (Table 6). This higher sorption capacity of woodchips when compared to soil may be attributed to higher organic carbon content and available macro and micropores [81]. Such a result is consistent with that obtained by of Valhondo et al. [48], who investigated the sorption capacities of various porous materials, including clay, zeolite, biochar, compost and woodchips. Among these materials, wood exhibited the highest sorption capacity for anionic compounds compared to sand. Nevertheless, such a retention is limited if compared to that described in the literature for other pharmaceuticals (more discussions are provided below). Indeed, calculated retardation factors range from 4.25 to 5.54, confirming the moderate effect of such a process.

The model reveals that, independently of the selected scenario, the simulated degradation rate constant μ_w_ in the woodchip layer is, as for *K_d_*, one to two orders of magnitude higher (up to 1.090 d^−1^) than that fitted for Column S (Table 6). The increase of this reactive parameter is very likely related to enhanced microbial activity as a consequence of the soil amendment rather than irreversible sorption processes [36,37]. However, irreversible sorption, believed by the authors to be a secondary process, cannot be quantified, since analyses of sorbed ketoprofen onto the porous material were not performed.

The column experiments were performed to explore if soil amendments using woodchips would have a positive impact on the wastewater treatment through VFs, while the modelling was developed to quantify the magnitude of the reactive processes assisting in data interpretation on the role of sorption and degradation. The domain mainly dominated by degradation processes corresponds to the plateau region of the breakthrough curves (Figure 5). Simulated values indicate a ketoprofen attenuation that remarkably increases from 54% (effluent concentration of 634.7 µg L^−1^) to 96.7–97.5% (effluent concentration of 25.0–32.6 µg L^−1^, respectively) when woodchips are incorporated as a layer over the soil surface. The obtained results are attributed to the effect of the microbial activity enhanced by the carbon source (from μ_w_ 0.058 d^−1^ to 1.082 d^−1^).

Comparisons with literature data in relation to the removal of ketoprofen when soil is amended with woodchips is anything but straightforward. To the authors´ knowledge, the scarce studies reporting the attenuation of this anti-inflammatory in the presence of woodchips are not comparable with our findings, whereas results from a few lab-scale experiments investigating the use of this material for removing other pharmaceuticals have been reported. For example, Ilhan et al. [81] have analyzed the sorption of the antibiotics enrofloxacin, monensin A and sulfamethazine in woodchips using bioreactors. The authors obtained values of *K_d_* for the pharmaceuticals in the range of 35 L kg^−1^ to 372 L kg^−1^ and concluded that the *K_d_* from monesin A and sulfamethazine obtained were higher than the values observed for the soil. The higher sorption of the analyzed compounds when compared to soil was attributed to the greater amount of organic matter provided by the woodchips.

Through columns filled with woodchips, Tseng et al. [82] studied the attenuation of five pharmaceuticals occurring in urban stormwater and concluded that removal rates strongly depend on the compound. Acetaminophen experiences an attenuation percentage greater than 80%, due to sorption and biodegradation processes, whereas the attenuation of ibuprofen is very limited (less than 15%), probably due to electrostatic repulsions with the negatively charged woodchip surface and to the anoxic conditions existing at the woodchip interface, which reduces the biodegradation for this chemical.

Experimental data along with numerical simulations clearly indicate that ketoprofen sorption and degradation are fostered by the presence of woodchips, and, from a process general point of view, our results coincide with those observed either by Ilhan et al. [81] or Tseng et al. [82] for other pharmaceuticals. However, when comparing the model quantified magnitude of such an effect with published data, the results were that sorption onto woodchips is rather limited, suggesting that degradation is the predominant attenuation mechanism.

Under the experimental pH (8.15 ± 0.20), ketoprofen was dissociated in the anionic form (pKa = 4), and sorption was likely hampered due to electrostatic repulsions both with the negatively charged soil and lignin, considered a crucial substance for the sorption of hydrophobic organic compounds in woodchips [83,84,85]. However, the carboxylate and keto-groups of ketoprofen can form complexes with surface metal species such as aluminum and iron, as well as metal cations like Al^3+^ and Fe^3+^ [86]. This phenomenon may account for the observed sorption onto the soil. Although ketoprofen has been reported as susceptible to degradation by fungi and bacteria under aerobic conditions [87,88,89], data quantifying such a process during infiltration through woodchips are not available in the literature. Therefore, the value fitted by our model cannot be quantitatively compared with published references.

### 3.4. Sensitivity Analysis

The resulting sensitivity coefficients computed for the input parameters for each range of perturbation are given in Table 7.

As shown in Table 7, the sensitivity coefficient of tested parameters in the woodchip layer varies from 0.014 to 22.682. The input parameters θ_smo_, α and n are identified as the most sensitive among the analyzed parameters. In contrast, the remaining parameters (θ_sIm_, θ_rmo_, θ_rIm_, l and ω) exhibit a marked lower sensitivity, whose associated uncertainty has a negligible impact on the transport model’s ability to replicate the experimental data.

The program Rosetta incorporated into Hydrus 1D has been developed specifically for soils and data required for the estimation of hydraulic parameters, among which θ_smo_, as well as α and n (empiric parameters in the soil water retention function) cannot be extrapolated for woodchips. In our study, we were able to calibrate woodchip θ_smo_, α and n by fitting flow experimental data and using Column S as a reference (leaving invariant previous fitted parameters for soil). Furthermore, θ_s_ was measured gravimetrically (0.85 cm^3^ cm^−3^) and partitioned between the mobile and immobile water content (θ_smo_ and θ_sim_) using inverse modelling.

As indicated by the sensitivity coefficients (see Table 7), the variation of these parameters has a moderate to low impact on simulated ketoprofen concentration (refer to Supplementary Materials, Figure S2). More specifically, the variation of θ_smo_, α or n has a greater impact on the rising limb of the breakthrough curve (average relative deviation of ketoprofen concentrations of 6.70%), whereas the influence on the peak ketoprofen concentration is relatively smaller, with an average relative deviation of 3.01%. This suggests that the variations in θ_smo_, α and n have a limited impact on the overall accuracy of the model in simulating the ketoprofen removal percentage in Column WS.

### 3.5. Qualitative Description of the Attenuation of Other Target Pharmaceuticals

Contrary to ketoprofen, the pharmaceuticals acetaminophen, atenolol, caffeine, carbamazepine, naproxen and sulfamethoxazole were never detected in Column S and Column WS effluents, indicating higher sorption and/or biodegradation of these compounds, independently of the presence of woodchips. With the exception of carbamazepine and sulfamethoxazole, such results corroborate the findings of Martínez-Hernández et al. [32] where the unamended soil of the VF is able to strongly attenuate (>90%) these pharmaceuticals in the pilot VF. The high biodegradability of acetaminophen and caffeine in biological treatments and natural environments such as soil has been already described [90,91,92,93,94,95].

On the other hand, many authors have reported carbamazepine to be a highly persistent pharmaceutical in the environment and frequently detected in surface and groundwater [96,97,98,99]. Indeed, the detection in groundwater already provides an approximation of its recalcitrant behavior. In soil, it is reported that sorption is the process dominating the transport of carbamazepine [35,100]. Due to its hydrophobic characteristics [101], the sorption is highly dependent on soil organic matter content [102,103], and biodegradation seems to play a secondary role during its transport through the unsaturated zone [35].

The lack of carbamazepine detection in any of the column experiments, despite it being a well-known recalcitrant contaminant, could be due to a strong sorption in a soil with a relatively high organic matter content (2.44%). Therefore, it is possible that the experiments were not long enough for the detection of the anticonvulsant in the columns’ effluents. However, although limited, an incomplete biodegradation cannot be ruled out, since low concentrations (up to 2.54 ug L^−1^) of the TP 10,11-epoxy carbamazepine were detected in Column WS’ effluent and remained relatively stable throughout the entire experiment. The stability of the concentration could be the result of the constant-rate transformation of the detected TP (transient intermediate) into a further product as suggested by Li et al. [104]. The absence of the TP in Column S likely confirms that the woodchips have an impact on the microbial activity able to partially biodegrade carbamazepine.

**Figure 6:**
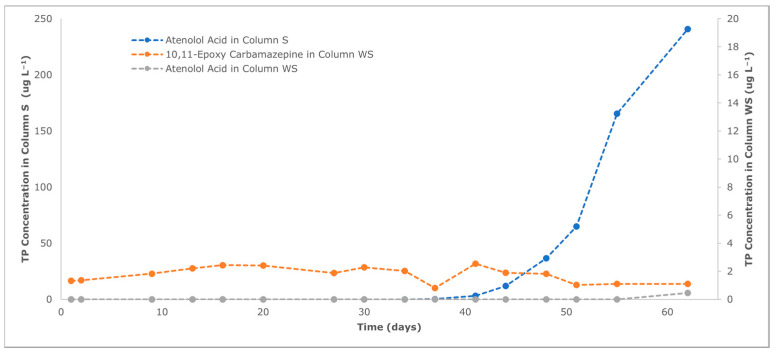
Detection of TPs of atenolol and carbamazepine at Column S and WS effluents. The secondary y axis corresponds to TP concentration detected at Column WS effluent.

Atenolol is positively ionized at the experimental pH (8.15 ± 0.20) (pKa of 14.08, as acid and 9.27 as base) and has a strong affinity for negatively charged soil particles. Therefore, interactions with soil inorganic surfaces, including cation exchange and electrostatic interactions, as well as with organic matter and clay materials, are the most relevant sorption mechanisms [105,106]. Given that the soil used for column experiments has a moderate cation exchange capacity (10.35 cmol_c_ kg^−1^), it is expected that atenolol will be moderately sorbed in Column S soil through cation exchange processes, as already obtained by other authors [105,106]. Furthermore, the formation of a major atenolol TP such as atenolol acid [103] with concentrations reaching 240.76 µg L^−1^ after 62 days of the experiment (see Figure 6) confirms that biodegradation of atenolol is also taking place. In Column WS, atenolol acid only begins to be detected towards the end of the experiment. This may indicate that atenolol: (i) is retained more strongly by sorption onto negatively charged woodchips and/or (ii) the TP atenolol acid is further degraded under the enhanced microbial activity conditions. In both cases, woodchips positively impact the attenuation of atenolol.

Naproxen is an amphiphilic molecule with a non-polar aromatic and anionic polar carboxylic acid functional groups [107]. Its log octanol-water partitioning coefficient (logK_ow_) of 3.18 and pKa of 4.24 indicates that the contaminant is relatively hydrophobic and negatively dissociated at the experimental pH of 8.15. The interactions with the soil organic matter should therefore predominate over sorption onto inorganic surfaces [106,107]. In terms of biodegradation, research findings suggest that naproxen’s attenuation is notably effective under aerobic and unsaturated conditions [35], being the predominant process during its transport in the unsaturated zone [95,108]. On the other hand, Zhang et al. [86] report on cooperative adsorption mechanisms of naproxen in the presence of other NSAIDs, including, but not limited to, ketoprofen, ibuprofen and diclofenac, in a mixed-compound system, akin to the conditions occurring in the experiment of this study. The authors observed a slightly lower *K_d_* for naproxen in a mixture-compound environment, suggesting competitive interactions with the other anionic NSAIDs for sorption active sites. However, the occurrence of such interactions cannot be either confirmed or ruled out when considering the results obtained in our experiment.

For sulfamethoxazole, biodegradation also seems to play an important role in its transport through soil as described by Lin et al. [109]. Sorption is expected to be secondary due to its negative charge under the experimental pH (pKa of 5.86, as acid and 1.97 as base). This biodegradation has been described as occurring under both aerobic [92] and anaerobic conditions [110]. However, Banzhaf et al. [100] and Barbieri et al. [111] suggest that the discrepancy in the behavior of sulfamethozaxole reported by available studies relies on the fact that its biodegradation is controlled by the dynamic between nitrate and nitrite during denitrification processes. Antibiotic concentration is rapidly depleted when nitrite builds up (nitrate reducing conditions) and increases again when nitrite is further reduced. As reported by Meffe et al. [36], nitrification/denitrification processes occurred in both columns, but the lack of sulfamethoxazole in the effluents did not allow the researchers to draw conclusions regarding the dependency of antibiotic behavior on nitrate and nitrite concentrations.

## 4. Conclusions

This study highlights the usefulness of modelling tools to evaluate the efficiency of pharmaceutical attenuation, not only in soil but also in more complex pore systems, such as woodchips, under variable saturated conditions. Column experiments coupled to reactive transport models allowed us to test soil amendment prior to its application in a VF and to identify and quantify the most important processes governing pharmaceutical attenuation. The flow modelling results indicated that a single-porosity (physical equilibrium) model was adequate to quantify soil layer hydraulic parameters, but a dual-porosity model was needed to simulate data when woodchips are incorporated, in order to obtain a more accurate representation of the water flow dynamics within this specific layer. Calibrated hydraulic parameters indicate that 67.9% of the woodchip pores are intra-aggregate immobile pores.

Ketoprofen is the only injected pharmaceutical that reached the column outlet of both systems within the experimental time. An equilibrium sorption and a first-order degradation model allow us to quantify the processes responsible for ketoprofen attenuation and to complete its breakthrough curves. The calibrated reactive transport parameters obtained when a woodchip layer is incorporated are about 1 to 2 orders of magnitude higher than the same parameters for the soil layer, indicating that the presence of woodchip in the soil columns increase the removal of this compound by enhancing the processes of sorption (81% in average) and, to a higher degree, biodegradation (913% in average). The fitted breakthrough curves provide an average removal of 54.0% of ketoprofen in soil and 96.7–97.5% when woodchips are incorporated.

The absence of acetaminophen, atenolol, caffeine, carbamazepine, naproxen and sulfamethoxazole in both columns’ outlets indicates that the compounds are attenuated independently of the woodchip amendment. However, the presence of the TPs 10,11-epoxy carbamazepine in Column WS and atenolol acid in both columns suggest that partial degradation also occurs. The effect of the amendment on the behavior of TPs is compound-specific. The formation of atenolol acid is reduced and/or its concentration further attenuated when woodchips are present, whereas 10,11-epoxy carbamazepine is detected only when soil is amended with woodchips, confirming that woodchips have an impact on the microbial activity and are able to partially degrade carbamazepine.

## Figures and Tables

**Table 1:** Model parameters.

Parameter			Values			Unit
			Column S	Column WS		
				Soil Layer	Woodchip Layer	
Model domain	Column characteristics	Profile length	18.00	28.00		cm
		Number of materials	1	2		-
		Layer thickness	18.00	18.00	10.00	cm
	Grid discretization	Number of nodes	1001	1001		-
		Number of fixed points for mesh density	3	3		
		Location of fixed points	1/557/1001	1/427/1001		
		Mesh upper density/lower density applied at fixed points	1:1/1 557:10/10 1001:1/	1:1/0.2 427:0.1/0.1 1001:0.3/1		-
		Number of observation points	3	3		-
		Location of observation points	1/220/644	1/736/1001		-
Time domain	Time discretization	Initial time step	0.1	0.1		s
		Minimum time step	0.01	0.01		s
		Maximum time step	1.728 × 10^7^	1.728 × 10^7^		s
		Simulation time	200.00	200.00		d
Hydraulic properties	Measured	Bulk density (ρ)	1.40	1.40	0.12	g cm^−3^
		Sand	55.00	55.00	-	%
		Silt	26.70	26.70	-	%
		Clay	18.30	18.30	-	%
		Saturated water content (θ_s_)	0.4207	0.4207	0.85	cm^3^ cm^−3^
	Rosetta estimation	Residual water content (θ_r_)	0.058	0.058	-	-
Hydraulic properties	Rosetta estimation	Empiric parameter in the soil water retention function (α)	0.0173	0.0173	-	cm^−1^
		Empiric parameter in the soil water retention function (n)	1.45	1.45	-	-
		Tortuosity parameter in the conductivity function (l) ^(a)^	0.50	0.50	-	-
Initial conditions	Flow ^(b)^	Upper pressure head	−18.00	−28.00		cm
		Lower pressure head	0.00	0.00		cm
	Transport	NaCl concentration	0.00	0.00		cm s^−1^
		Pharmaceutical concentration	0.00	0.00		cm s^−1^
Boundary conditions	Flow	Tracer flux (1 s single pulse)	0.6367	0.6367		cm s^−1^
		Pharmaceutical flux (pulse: 1 s every 24 h)	0.6367	0.6367		cm s^−1^
		Maximum h at the soil surface	0.65	0.65		cm
	Transport	NaCl concentration	0.1711	0.1711		mmol cm^−3^
		Daily pharmaceutical concentration	1.00	1.00		mg L^−1^
Calibration parameters	Woodchip hydraulic parameters	Mobile residual water content (θ_rmo_)	X	X	X	cm^3^ cm^−3^
		Mobile saturated water content (θ_smo_)			X	cm^3^ cm^−3^
		Empiric parameter in the soil water retention function (α)	X	X	X	cm^−1^
		Empiric parameter in the soil water retention function (n)	X	X	X	-
		Immobile residual water content (θ_rim_)			X	cm^3^ cm^−3^
		Immobile saturated water content (θ_sim_)			X	cm^3^ cm^−3^
Calibration parameters	Woodchip hydraulic parameters	Mass transfer coefficient (ω)			X	s^−1^
	Tracer transport	Saturated hydraulic conductivity (K_s_)	X	X	X	cm s^−1^
		Longitudinal dispersivity (α_L_)	X	X	X	cm
		Tracer molecular diffusion coefficient in free water (D_w_)	X	X	X	cm^2^ s^−1^
	Pharmaceutical transport	Distribution coefficient (K_d_)	X	X	X	cm^3^ g^−1^
		First-order kinetic removal rate (μ_w_)	X	X	X	s^−1^
		Pharmaceutical molecular diffusion coefficient in free water (D_w_)	X	X	X	cm^2^ s^−1^

^(a)^ Mualem [59]. ^(b)^ defined in the model domain as the linear progression between upper and lower pressure head.

**Table 2:** Cumulative experimental and simulated water volumes flowing out from the columns’ outlets.

Parameter	Column S	Column WS	
	Single-Porosity Model (mL)	Single- Porosity Model (mL)	Dual- Porosity Model (mL)
Cumulative experimental water volume	2032.34	2016.97	2016.97
Cumulative simulated water volume	2033.61	1937.87	1995.72
Root Mean Squared Error (RMSE)	4.59	9.40	4.95

**Table 3:** Ks and α_L_ of the soil and woodchip layers obtained though the inverse fitting of the tracer transport model.

Parameter	Soil Layer	Woodchip Layer
K_s_ (cm s^−1^)	3.11 × 10^−5^	0.0218
α_L_ (cm)	0.0696	0.748

**Table 4:** Woodchip hydraulic parameters obtained though inverse fitting of Cl^−^ concentrations obtained from the tracer test in Column WS.

Parameter	Value
	Woodchip Layer
θ_rmo_ (cm^3^ cm^−3^)	1.52 × 10^−4^
θ_rIm_ (cm^3^ cm^−3^)	0.00
θ_smo_ (cm^3^ cm^−3^)	0.273
θ_sIm_ (cm^3^ cm^−3^)	0.577
α (cm^−1^)	0.02
n (-)	1.50
ω (s^−1^)	3.54 × 10^−7^

θ_rmo_ is the residual water content for the woodchip mobile region; θ_rIm_ is the residual water content for the woodchip immobile region; θ_smo_ is the saturated water content for the woodchip mobile region; θ_sIm_ is the saturated water content for the woodchip immobile region; α is an empirical parameter of the water retention function; n is an empirical parameter of the water retention function; ω is the woodchip first-order mass transfer coefficient.

**Table 5:** Column S simulation results from applying a variation of ±20% in reactive transport parameters.

Scenario	*K_d_* (L kg^−1^)	µ_w_ (d^−1^)	R^2^	RMSE (μg L^−1^)	Parameter Variation (%)	
					*K_d_*	µ_w_
Best fit model	0.482	0.0729	0.991	12.64	-	-
*K_d_* + 10%	0.530	0.0647	0.979	20.49	10.0%	−11.3%
*K_d_* + 20%	0.579	0.0563	0.953	31.75	20.0%	−22.8%
*K_d_* − 10%	0.434	0.0802	0.986	15.67	−10.0%	9.9%
*K_d_* − 20%	0.386	0.0867	0.960	26.87	−20.0%	18.9%
µ_w_ + 10%	0.452	0.0802	0.990	16.02	−6.4%	10.0%
µ_w_ + 20%	0.423	0.0875	0.980	24.54	−12.2%	20.0%
µ_w_ − 10%	0.514	0.0657	0.986	16.93	6.6%	−10.0%
µ_w_ − 20%	0.547	0.0584	0.970	24.51	13.5%	−20.0%

**Table 6:** Calibrated sorption and degradation parameters for soil and woodchip layers in Column WS.

Model	Parameter	Soil Layer	Woodchip Layer	R^2^	RMSE
Scenario 1	*K_d_*	0.482	13.110	0.997	0.494
	μ_w_	0.255	0.255		
Scenario 2	*K_d_*	0.482	15.260	0.997	0.506
	μ_w_	0.092	0.709		
Scenario 3	*K_d_*	0.482	11.200	0.997	0.567
	μ_w_	0.379	0.000		
Scenario 4	*K_d_*	0.482	15.410	0.995	0.526
	μ_w_	0.000	1.090		
Scenario 5	*K_d_*	0.482	15.250	0.996	0.523
	μ_w_	0.073	0.783		

*K_d_*: linear isotherm adsorption coefficient expressed in L kg^−1^; μ_w_: first-order kinetic removal rate expressed in d^−1^; R^2^: correlation coefficient between observed and simulated data; RMSE: root mean squared error; Scenario 1: similar μ_w_ in the soil and woodchip layers; Scenario 2: different μ_w_ in the soil and woodchip layers; Scenario 3: μ_w_= 0 in the woodchip layer; Scenario 4: μ_w_= 0 in the soil layer; Scenario 5: same μ_w_ and *K_d_* as Column S in the soil layer of Column WS.

**Table 7:** Results from the sensitivity analysis. The absolute values are shown.

Layer	Parameter	% Change in Parameter	Sensitivity Coefficient
Woodchips	θ_sIm_	−25%	0.122
		−15%	0.111
		15%	0.125
		25%	0.043
	θ_smo_	−25%	1.242
		−15%	0.973
		15%	22.682
		25%	2.334
	θ_rmo_	−25%	0.034
		−15%	0.102
		15%	0.033
		25%	0.055
	α	−25%	0.110
		−15%	0.198
		15%	5.625
		25%	5.054
	n	−25%	1.404
		−15%	3.019
		15%	1.679
		25%	1.911
	l	−25%	0.014
		−15%	0.169
		15%	0.094
		25%	0.035
	ω	−25%	0.125
		−15%	0.080
		15%	0.061
		25%	0.023

## Data Availability

The raw data supporting the conclusions of this article will be made available by the authors on request.

## Supplementary Materials

The following supporting information can be downloaded at: https://www.mdpi.com/article/10.3390/toxics12050334/s1, Figure S1: Experimental and corresponding simulated average outflow flow rates from Column WS applying a single-porosity model (RMSE: 0.030 mL/min). Figure S2: Column WS simulated ketoprofen breakthrough curves resulting from the base model run and from the sensitivity analysis when (a) α, (b) n and (c) θ_smo_ are varied ±25%.
