# Supplementary material for: Amended Vegetation Filters as Nature-Based Solutions for the Treatment of Pharmaceuticals: Infiltration Experiments Coupled to Reactive Transport Modelling
Source: Toxics. 2024 May 4;12(5):334. doi: 10.3390/toxics12050334 (PMC11125991; doi:10.3390/toxics12050334)
Supplement: Supplementary file 1 [file toxics-12-00334-s001.zip › toxics-2941789-supplementary.pdf]

# Amended Vegetation Filters as nature-based solutions for the treatment of pharmaceuticals: infiltration experiments coupled to reactive transport modelling – Supplementary Materials

## A) Column WS average flow rate simulated with the single-porosity model

**Figure S1** presents experimental and simulated average flow rates when a single-porosity approach is applied in Column WS.

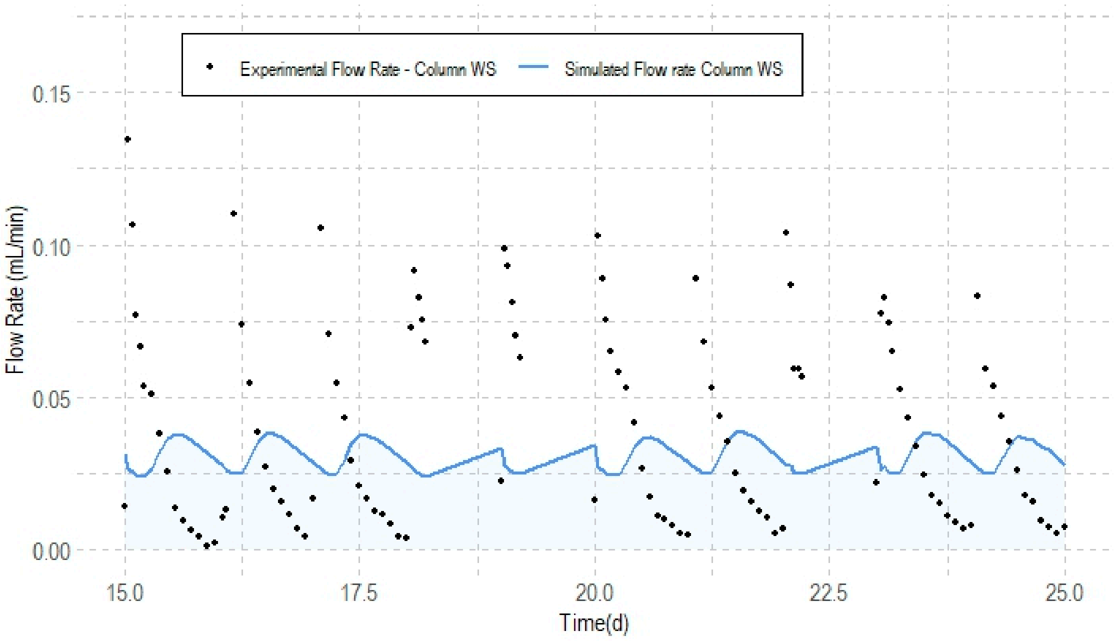


**Figure S1.** Experimental and corresponding simulated average outflow flow rates from Column WS applying a single-porosity model (RMSE: 0.030 mL/min).

## B) Sensitivity Analysis: Effect on simulated ketoprofen breakthrough curves

**Figure S2** presents the Column WS ketoprofen breakthrough curves resulting from the base model run and from the sensitivity analysis when $\theta_{smo}$, $\alpha$ and n are varied ±25%.

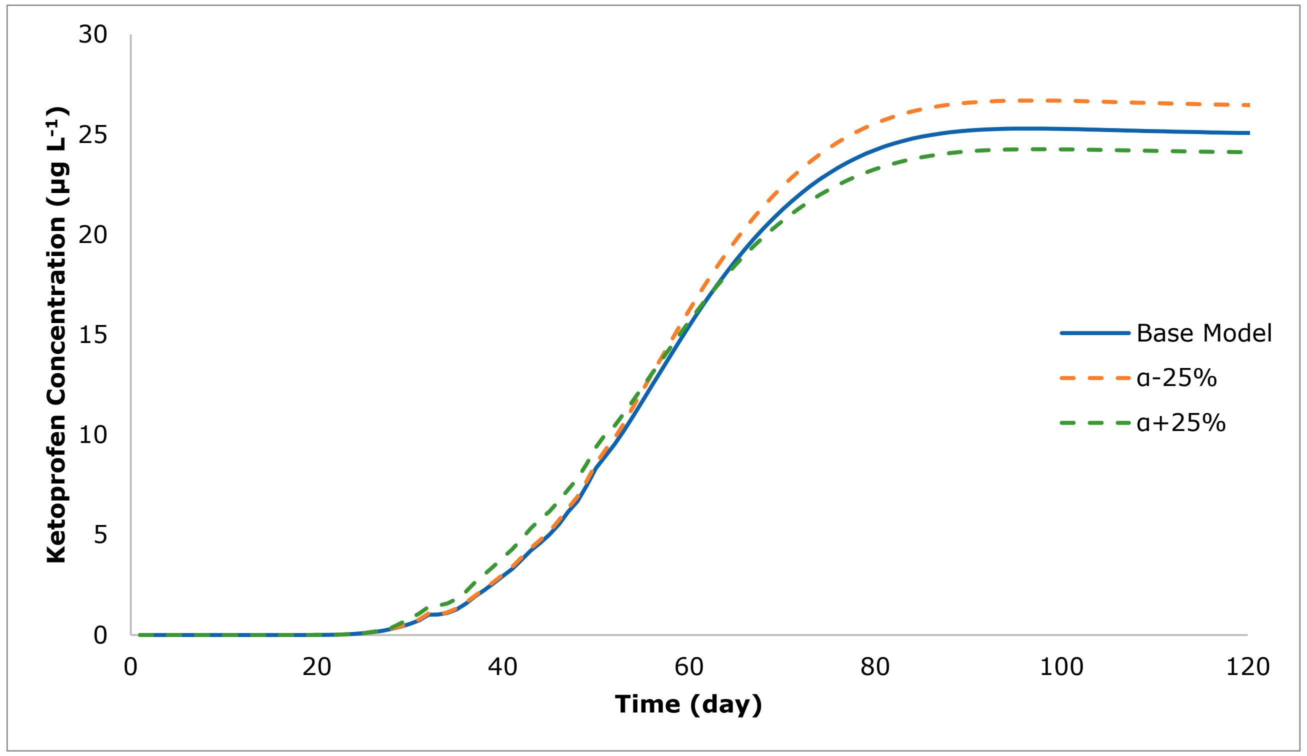


**(a)**

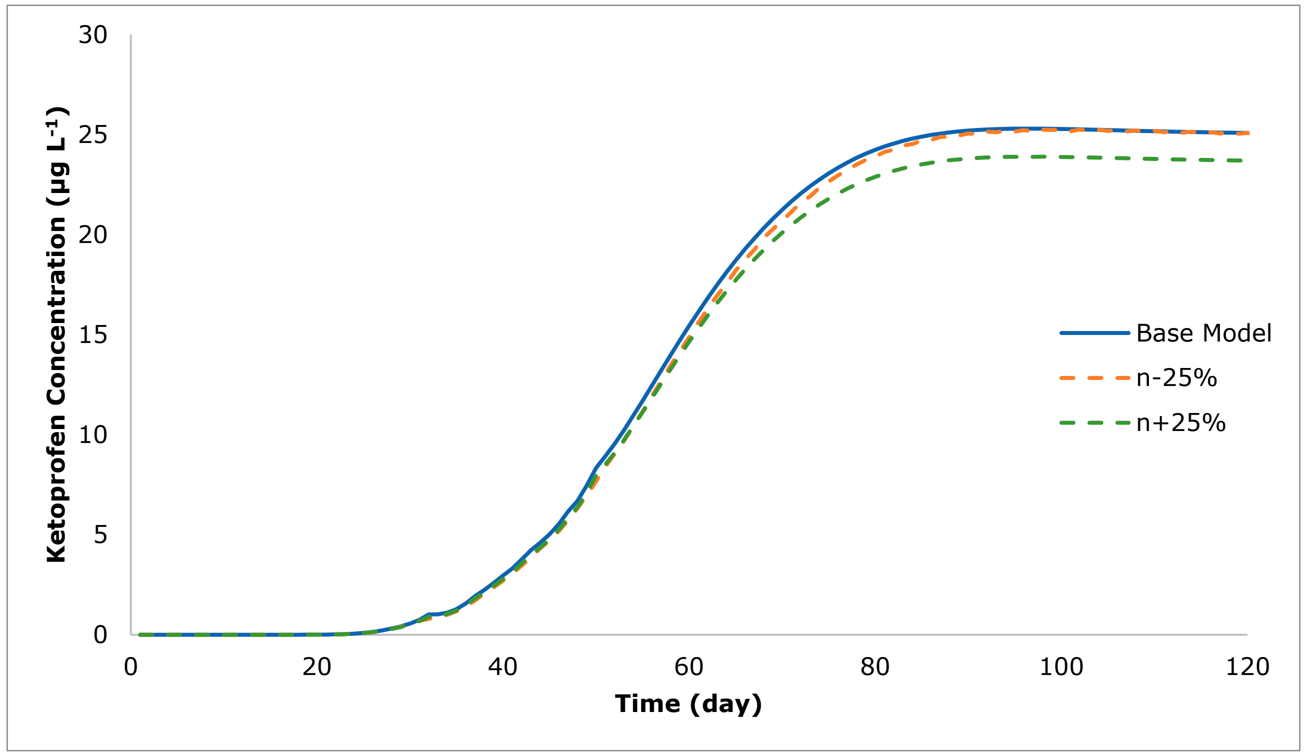


**(b)**

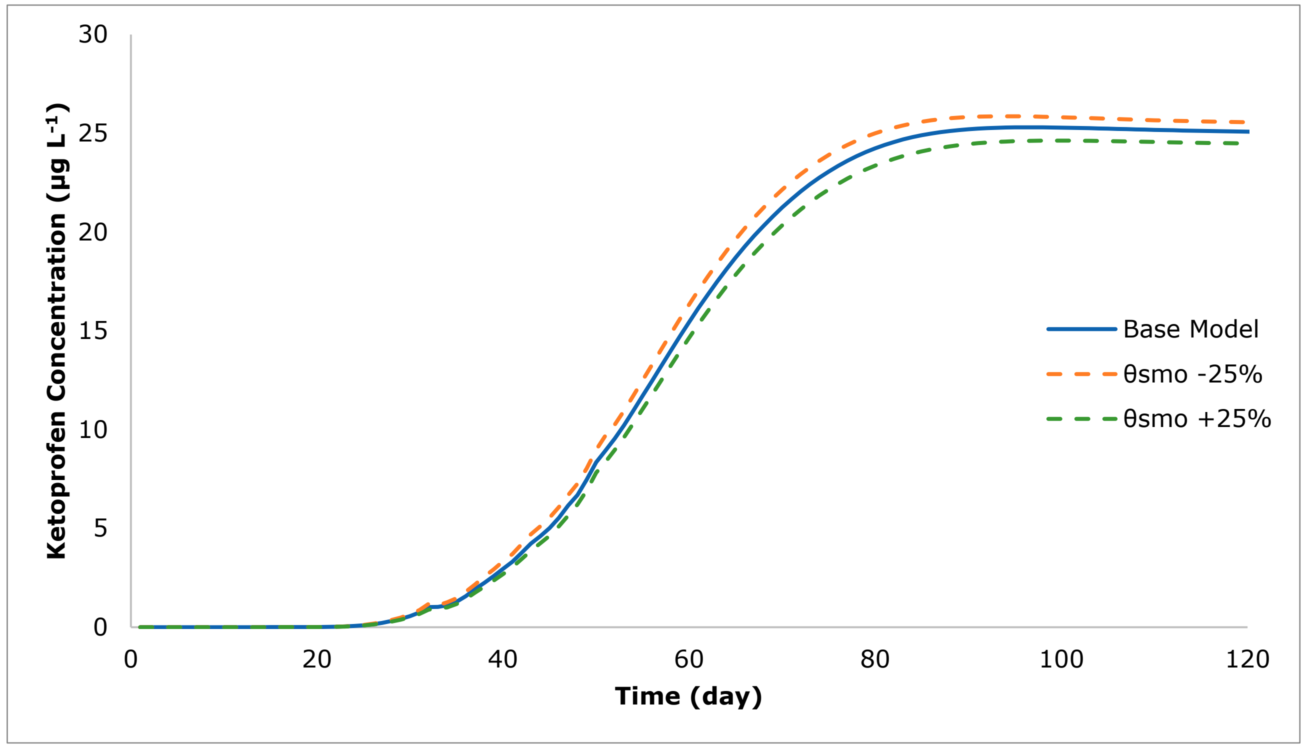


**(c)**

**Figure S2.** Column WS simulated ketoprofen breakthrough curves resulting from the base model run and from the sensitivity analysis when (a) $\alpha$, (b) n and (c) $\theta_{smo}$ are varied ±25%.
